# Glucosinolates Mediated Regulation of Enzymatic Activity in Response to Oxidative Stress in *Brassica* spp.

**DOI:** 10.3390/plants13233422

**Published:** 2024-12-05

**Authors:** Aishmita Gantait, Sam A. Masih, Rosangela Addesso, Ann Maxton, Adriano Sofo

**Affiliations:** 1 Department of Genetics and Plant Breeding, Sam Higginbottom University of Agriculture, Technology and Sciences, Prayagraj 211007, India; aishmitagantait@gmail.com; 2 Department of Molecular and Cellular Engineering, Sam Higginbottom University of Agriculture, Technology and Sciences, Prayagraj 211007, India; sam.masih@shiats.edu.in; 3 Department of Agricultural, Forestry, Food and Environmental Sciences (DAFE), University of Basilicata, 85100 Potenza, Italy; rosangela.addesso@unibas.it

**Keywords:** antioxidants, *Brassica*, glucosinolates, oxidative stress, ROS

## Abstract

*Brassica* crops are vital as they supply essential minerals, antioxidants, and bioactive substances like anthocyanins, glucosinolates, and carotenoids. However, biotic and abiotic elements that cause oxidative stress through heavy metals and other eco-toxicants pose a risk to *Brassica* plants. Increased generation of Reactive Oxygen Species (ROS) causes oxidative stress, which damages biomolecules and interferes with plant growth, productivity, and cellular equilibrium. Plants producing *Brassica* need an intricate enzyme defence mechanism to fend off oxidative stress. All the enzymes that have been addressed are found in mitochondria, peroxisomes, chloroplasts, and other cell components. They are in charge of removing ROS and preserving the cell’s redox balance. Additionally, *Brassica* plants use secondary metabolites called Glucosinolates (GLs), which have the capacity to regulate enzymatic activity and act as antioxidants. By breaking down compounds like sulforaphane, GLs boost antioxidant enzymes and provide protection against oxidative stress. To develop methods for improving agricultural crop stress tolerance and productivity in *Brassica,* it is necessary to comprehend the dynamic interaction between GL metabolism and enzymatic antioxidant systems. This highlights the possibility of maximizing antioxidant defences and raising the nutritional and commercial value of *Brassica* across the globe by utilizing genetic diversity and environmental interactions.

## 1. Introduction

*Brassica* crops, such as *B. oleracea*, *B. napus*, and *B. juncea*, are fundamental to agriculture worldwide. Because of their high nutritional value, being rich in essential minerals, antioxidants, and bioactive compounds (e.g., carotenoids, glucosinolates and anthocyanins), these crops support human health and ensure food security [1]. Thanks to the highly diverse genetic pool of *Brassica* species, breeding efforts aimed at creating superior high-yielding and climate-tolerant crop varieties have benefitted greatly, with relevant impacts in agriculture, food production, and the global economy. Rapeseed-mustard is one of India’s main oilseed crops, and *Brassica* crops are essential for oil extraction [2]. It was estimated that 71.24 million metric tons of rapeseed will be produced globally, with the major producers being the European Union, Canada, China, and India [3]. With an area of 6.23 million hectares and a productivity of 1499 kg/ha, rapeseed-mustard accounts for 28.6% of India’s total oilseed production [4]. It is mainly cultivated in the states of Punjab, Rajasthan, Uttar Pradesh, and Madhya Pradesh. By 2030, the country is expected to contribute 16.4–20.5 million metric tons of oilseeds, highlighting the necessity of increasing rapeseed-mustard production to meet the growing demand [5].

Oxidative stress in eukaryotic cells represents an imbalance between the production of Reactive Oxygen Species (ROS) and the antioxidant defense system that can lead to cellular damage and the potential onset of disease [6]. In *Brassicaceae* family (including *Brassica juncea* and *Cakile maritime*), exposure to harmful trace metal elements, such as barium (Ba), triggers oxidative stress reactions that activate antioxidant defense mechanisms, such as guaicol peroxidase, ascorbate peroxidase (APX), and catalase (CAT) [7]. Additionally, some species, to counteract the consequences of increased ROS generation, increase the production of secondary metabolites such as flavonoids and total phenols, whereas others have a two-celled structure that allows them to thrive in conditions with elevated Ba concentrations [7]. These traits demonstrate how plants can use both enzymatic and non-enzymatic ROS transformation pathways to control oxidative stress and prevent cellular damage; this is particularly true in *Brassica* plants, including the most consumed cauliflowers, broccoli and cabbages. Glucosinolates (GLs) are essential secondary metabolites found in *Brassica* plants, and they are useful for controlling enzyme activity in response to oxidative stress. GLs are phytochemicals that are known for their antioxidant qualities and doubled haploid lines (DHLs). They are especially prevalent in *B. rapa*, which has high GLs content (HGSL) and was intentionally developed from two edible subspecies of *B. rapa*: subsp. *trilocularis* and *B. rapa* subsp. *chinensis* [5]. As chemicals, they offer defense against biotic stresses. Through hydrolysis, GLs produce isothiocyanates, which have beneficial effects on antioxidants as they activate cellular protection systems, lessen oxidative burden, and improve mitochondrial efficiency and protein stability during muscle strain, thus reducing cellular lesions during workouts [1]. Experimental work has shown that GLs like Sinigrin (SIN) can change their content depending on the environmental stress, accumulating when the plant is exposed to salt [8]. Additionally, it has been demonstrated that GL breakdown products can affect cellular functions through decreased oxygen consumption, increased ROS buildup, and fungal oxidative stress response gene regulation. Furthermore, the GL derivative indole-3-carbinol has been found to protect against oxidative DNA damage by activating the aryl hydrocarbon receptor (AhR) pathway, suggesting a new role for GLs in oxidative stress defense mechanisms [9]. Therefore, GLs work in a multifaceted way in controlling enzymatic reactions due to oxidative stress in *Brassica* plants, pointing to the larger roles they play in defense and adaptation.

## 2. Oxidative Stress and Associated Enzymes

Oxidative stress is a condition that occurs when ROS are produced in overabundance, or when the antioxidant defenses are impaired or over helmed. As a result, excess ROS interact with cell macromolecules, damaging them. ROS are the highly reactive molecules produced in plants cells, especially during abiotic stress. In plant cells, ROS can be generated in organelles, such as chloroplasts, mitochondria, and peroxisomes [10]. For instance, chlorophyll, acting as a light-absorbing center, can lead to thr formation of ^1^O_2_ [11]. This singlet oxygen may spread to other bodily cellular compartments, leading to harmful effects. Additionally, the mitochondrial electron transport chain and nicotinamide adenine dinucleotide phosphate (NADPH) oxidases produce superoxide, thereby increasing the cell’s normal ROS burden. When atmospheric oxygen (O_2_) is in a stable triplet state (^3^O_2_) with two unpaired electrons, ROS are produced [12]. This state limits its reactivity. However, when energy is invested through biochemical reactions, such as electron carrier transport (Electron transport chain) or through exposure to physical stimuli like UV light, 3 molecule O_2_ switches to a reactive excited state, ^1^O_2_ and O_2_•^−^. Some of the most studied ROS include the superoxide anion (O_2_•^−^), hydrogen peroxide (H_2_O_2_), and the hydroxyl radical (•OH) [13]. Each one has unique properties and reactivity. For instance, O_2_•^−^ can react with protons (H+) to form hydroperoxyl radicals (HO_2_•^−^), which are more reactive and can easily permeate biological membranes. ROS not only act as damaging agents but also as signalling molecules [14]. Through cellular signaling, they can influence the oxidative stress regulation, the growth and the development of plant cells, as well as their antioxidant defense mechanisms. Moreover, they can trigger numerous biological processes, such as stress adaptation or defense mechanisms. For instance, they control redox signaling, activating the Mitogen-Activated Protein Kinase (MAPK) pathway and increasing plant resistance to biotic and abiotic stressors [15]. The delicate interplay between ROS generation and neutralization is a key challenge. ROS in plants are mostly detoxified by non-enzymatic antioxidants, such as CAT and SOD. To prevent oxidative damage and the consequent cell dysfunction and damage, this equilibrium is crucial [16].

Oxidative stress occurs when antioxidant defenses in biological systems fail to stabilize tissue oxidative processes, resulting from the reduction of molecular oxygen to form free radicals (oxidants), reactive metabolites with reducing potential, and other oxidizable substrates [17]. It throws off the balance between the production of reactive oxygen species, or free radicals, and antioxidant defenses [18]. In plants and other organisms, reducing oxidative stress is largely dependent on enzymatic control. As a result of physiological metabolism, ROS build up under biotic and abiotic stress conditions, causing oxidative damage and eventual cell death [19]. This imbalance in redox equilibrium damages biomolecules, including proteins, lipids, and nucleic acids [20]. It can result in heritable DNA changes, alterations or elimination of cell characteristics, and modifications in membrane permeability and in catalytic activity [21]. Lastly, these changes impair plant development and growth, lowering agricultural yield. In response to oxidative stress, plants activate their enzymatic (such as CAT and SOD) and non-enzymatic (such as carotenoids and phenolics) antioxidant systems to combat ROS, minimizing cellular damage, protecting macro- and micromolecules, and averting cell death [22]. Therefore, plants adapted to unfavourable environment conditions maintain an optimal rate of ROS generation and detoxification.

This highlights the crucial role of antioxidant defence systems in protecting plants against oxidative stress and ensuring health and productivity [23]. The enzymatic defence mechanism against ROS includes enzymes like glutathione peroxidase, peroxidase, SOD, polyphenol oxidase, APX, and CAT [24]. Some of these enzymes are used by plants to overcome stress situations and to stabilize redox processes. Furthermore, oxidative stress can directly alter the guanine-rich sequence in cancer-associated genes, with negative consequences on gene stability and function. The base excision repair pathway regulates gene expression through the OGG1, NEIL1-3, and APE1/REF1 enzymes [25]. Consequently, this pathway can either increase or reduce gene expression according to the location and conditions of the injury. Oxidative stress significantly affects *Brassica* plants by altering their antioxidant defense systems and physiological reactions [26]. The antioxidants causing oxidative burst, lipid peroxidation, and pigment content decrease in *Brassica* species are harmed by heavy metal contamination, such as that caused by arsenic and chromium, with detrimental effects on plant growth and development [27]. Additionally, the Ascorbic Acid-Glutathione cycle is upset by hazardous trace metals, such as lead and arsenic, which leads to oxidative stress and aberrant plant growth in *Brassica* [28]. In particular, a study has demonstrated that exposing *Brassica juncea* seedlings to chromium-induced stress, combined with rhizobacteria and earthworm applications, enhances the antioxidant defense system, with a subsequent decrease in ROS levels and improved plant biomass [29].

As for *Brassica* plants, higher levels of glucosinolates improve their ability to combat oxidative stress, which is an external environmental stress [30]. For instance, research has shown that plants containing higher levels of glucosinolate are less prone to oxidative stress in conditions of diseases or drought. Compared with their parent compounds, their breakdown products must have a protective role. Studies in *B. napus* (rapeseed) and *B. oleracea* (cabbage) revealed that when the GL level is high during stressful conditions, antioxidant enzyme activity and ROS levels were also high [26].

## 3. Enzymatic Defence Mechanism to Prevent Oxidative Stress

**Figure 1:**
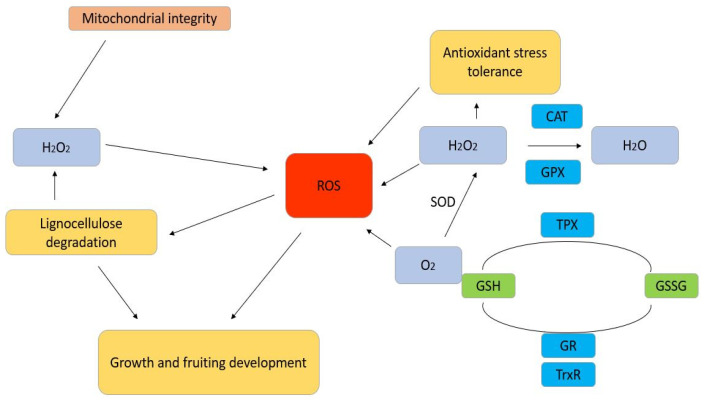
Enzymatic defence mechanisms to prevent oxidative stress in plants.

Plant enzymatic defence systems play a key role in ROS management and in oxidative tension decrease, thereby mitigating the effects of oxidative stress [30]. This antioxidant defense system involves enzymes such as Superoxide dismutase (SOD), Catalase (CAT), Ascorbate peroxidase (APX), Glutathione reductase (GR), Monodehydroascorbate reductase (MDHAR) and Dehydroascorbate reductase (DHAR), Glutathione peroxidase (GPX), and Peroxidase (POX) [31]. Collectively, these enzymes regulate ROS levels in plant cells, forming a network that enable cellular redox state maintenance under stress conditions [32] (Figure 1).

The various processes and conformations of enzymatic antioxidants make them crucial for scavenging ROS. These enzymes, including Glutathione Peroxidase, Catalase, and SOD, are pivotal in controlling the intracellular redox state and, consequently, in protecting cells from oxidative damages. For instance:

- SOD: It neutralizes superoxide radicals (O_2_•^−^), a singlet oxygen, and splits it into hydrogen peroxide (H_2_O_2_) and molecular oxygen (O_2_), depending on the presence of metal cofactors, such as Mn, Cu, or Zn, in its active site [33].
- Catalase: It promotes the breakdown of hydrogen peroxide in water and oxygen, which in turn decreases the amount of oxidative injury [34].
- Glutathione Peroxidase: It catalyzes the breakdown of hydrogen peroxide and organic peroxide using glutathione as a substrate [35].

To protect plants from oxidative stress, the Asada–Halliwell pathway—also known as the ascorbate-glutathione cycle—affects ROS regulation. Enzymes called APX and GR are crucial for preserving redox equilibrium and providing protection under various stressors. Therefore, evidence shows that the Asada–Halliwell pathway is active in *B. juncea* under chromium (Cr) stress, enhancing the activities of antioxidative enzymes, including APX and GR. These enzymes assist in oxidative stress decrease and in the enhancement of the plant’s ability to withstand metal toxicity [36]. Each element in the process, including glutathione and ascorbate, is crucial for controlling redox equilibrium. Under stress, cells accumulate such antioxidants to support the development and the safeguarding of their functions. For instance, increased APX and GR activities were observed responses to saline stress, confirming the pathway’s adaptability [37]. Additionally, the Asada–Halliwell pathway interacts with hormonal modulation, specifically abscisic acid (ABA) and jasmonic acid (JA), to control stress in *B. juncea* plants. These hormones regulate pathogenesis-related genes, showing a vast hormonal interplay in coordination with stress and antioxidant genes [38].

### 3.1. Superoxide Dismutase (SOD)

SOD is located in plant cells, where it helps to avoid oxidative damage and ensure the overall health and longevity of the plant. It is an essential enzyme that protects *Brassica* species from oxidative stress by removing ROS in adverse environments [39,40]. In plant cells, SODs serve as the main defence against ROS [41]. SODs are present in various compartments in plant cells, including the peroxisomes, mitochondria, and chloroplast, being the organelles involved in ROS generation [42]. By converting ROS into oxygen, hydrogen peroxide, or other non-destructive molecules, they mitigate possible dangers [43]. SOD has a crucial role in maintaining the balance between ROS production and removal, particularly during stressful situations when ROS levels rise [44]. Their genetic makeup and subcellular location dictate SOD’s specialized function, allowing efficient ROS detoxification across different cellular compartments [45]. Research has demonstrated that when sodium nitroprusside, a nitric oxide donor, is applied to *B. juncea*, it significantly improves the activity of antioxidative enzymes such as SOD, catalase, and peroxidase, improving salt tolerance and lowering oxidative damage caused by NaCl stress [46]. Genome-wide investigations in *B. rapa* and *B. junce* have identified SOD genes that react to abiotic stressors, such as heat, salinity, and drought, providing resistance mechanisms against these stresses [47]. Additionally, it has been shown that nitric oxide and antioxidants like SOD work together to reduce the oxidative stress brought on by NaCl, increasing the salt stress tolerance in *B. juncea* plants [48]. Furthermore, the application of 28-homobrassinolide before seeding boosts SOD and other antioxidant enzymes, hence lowering the oxidative stress caused by the high temperatures in *B. juncea* [49].

### 3.2. Catalase (CAT)

Catalases, core ROS-associated proteins, first appeared around 2.5 billion years ago and played a key role in the Great Oxidation Event [50]. CAT is essential for preventing oxidative stress in *Brassica* species by converting H_2_O_2_ into H_2_O [51]. This process reduces the ROS buildup, which could otherwise limit the growth and development of plants. Consequently, CAT enzymes promote the maintainance of cellular and organismal homeostasis by converting H_2_O_2_ into oxygen and water. This process decreases the level of oxidative injury and allows the growth and survival of plants under more severe conditions [52]. Recent studies have revealed that the rapeseed CAT gene family consists of 14 genes, and the exposure to various stresses, such as cold, salt, and hormone stress, significantly increases the expression of some of these genes [53]. Furthermore, in non-heading Chinese cabbage, overexpression of the BcWRKY22 gene increases CAT enzyme activity and improves thermotolerance, thereby reducing H_2_O_2_ buildup and confirming the direct link between CAT and boosting plant heat stress tolerance [54]. The importance of CAT in lowering oxidative damage under stress was also demonstrated by the application of β-aminobutyric acid (BABA) to *B. napus* under drought stress, which decreased lipid peroxidation, enhanced non-enzymatic antioxidants, and decreased H_2_O_2_ levels [55]. Additionally, *B. juncea* plants treated with 28-homobrassinolide prior to planting showed enhanced development under extreme temperature stress, reduced oxidative stress, and elevated CAT activity, all of which helped to maintain antioxidant capacity [56]. *Brassicaceae* and monocots have been shown to contain specific amino acid residues such as Cys-343 and Thr-343, which affect the functional variety of CAT genes in these plant families [57]. The influence of important amino acid residues on the catalytic capabilities and structural features of CAT proteins was determined using structural predictions and sequence alignments [58]. The significance of essential amino acids in regulating the CAT genes’ activities in various plant species was supported by the presence of conserved motifs and specific residues to individual plant species [59].

### 3.3. Ascorbate Peroxidase (APX)

To fight oxidative stress, or ROS, plant cells use both enzymatic and non-enzymatic substances, such as ascorbate and glutathione [60]. APX reduces oxidative stress by scavenging ROS in plants, including H_2_O_2_ [61]. APX reduces the levels of harmful ROS in plant cells by employing reduced ascorbate as an electron donor to convert H_2_O_2_ into water [62]. Different gene families in plants express distinct APX isoforms based on the subcellular compartment in which they are located [63]. There can be several isoforms of APX depending on the site where they exist, and these include Cytosolic APX, Mitochondrial APX, Chloroplast APX, and Peroxisomal APX [64]. By regulating the amount of ROS in cells and organelles, these isoforms protect plants from stressors and promote their growth [65]. The subcellular location of the plant APX isoenzyme is determined by the presence of transmembrane domains and organelle-specific targeting peptides [66]. Maintaining appropriate ascorbate levels is crucial for effective ROS elimination, since the APX isoenzymes contain bound ascorbate, and the increase of this antioxidant has been shown to change the stability and activity of these enzymes [67]. Beyond its conventional role as an ascorbate peroxidase, APX has also been demonstrated to have greater substrate selectivity and chaperone activity, hence augmenting its participation in a variety of biological processes [68]. *Brassica* APX genes, such as BnaAPX and BrAPX, are differently expressed under diverse conditions of stress, including heat, salt, drought, and cold, implying that these genes are very important as part of stress reactions [69]. According to research, *B. juncea* tolerance to salt stress is increased when APX genes are overexpressed because this increases the antioxidative defense mechanisms [70]. Additionally, added exogenous ascorbate (AsA) would improve the AsA–GSH–NADPH cycle’s ability to reduce ROS generation and strengthen the antioxidant defense system when *Brassica napus* is under Cd stress [71].

### 3.4. Glutathione Reductase (GR)

GR has a special role in mitigating oxidative stress in *Brassica* species via managing the antioxidant protection and oxidative status [72]. GR, an enzyme well known for its ability to neutralize oxidative stress and preserve cellular redox balance, is essential to *Brassica*’s antioxidant defense system [15]. GR genes (BcGR1.1, BcGR1.2, BcGR2.1, and BcGR2.2) are expressed in a range of tissues and are triggered by abiotic stressors such as cold, high temperatures, drought, salt stress, and Cd exposure, which raise GR gene expression and enzyme activity, according to research on *Brassica* species, including *Brassica campestris* and *Brassica napus* [57,73]. It has been demonstrated that overexpressing *Brassica rapa*, GR, in yeast and *E. coli* systems enhances cellular glutathione homeostasis, increases the activity of antioxidant enzymes, and increases resistance to oxidative stressors like exposure to H_2_O_2_ [74]. Furthermore, it was discovered that arsenic-induced stress significantly increased GR activity in Indian mustard (*B. juncea*), demonstrating the importance of the Ascorbate-Glutathione pathways in protecting against harmful compounds [75]. Glutathione (GSH), a GR substrate, can be applied exogenously to improve redox control and antioxidant defences against oxidative damage brought on by stressors such as Cd [76]. Furthermore, *Saccharomyces cerevisiae* that have overexpressed GR have an improved cellular redox equilibrium, which increases tolerance to oxidative stress brought on by a variety of stressors, including heat shock, heavy metals, and H_2_O_2_ [77]. In conclusion, by controlling redox balance and antioxidant systems, GR and its relationship with GSH are important in shielding *Brassica* plants from oxidative stress.

### 3.5. Monodehydroascorbate Reductase (MDHAR)

MDHAR impacts *Brassica’*s antioxidant defense by providing cytosol-specific isoforms, crucial for ascorbate recycling, a process essential for antioxidant function in *Brassicaceae* plants [78]. Due to its role in the regeneration of ascorbate, a vital antioxidant molecule, MDHAR is an essential component of *Brassica* plants’ antioxidant defense system [79]. In *Brassica* species, studies show that MDHAR can reduce oxidative damage brought on by heavy metals, including Pb and Cd [80]. In response to Cd stress in *Brassica* plants, MDHAR accumulates concurrently with other antioxidant enzymes, reducing oxidative damage and improving growth character, as was well-substantiated in many investigations [71]. Monodehydroascorbate Reductase activity decreased in all *Brassica* species under Cd stress with the exception of *B. juncea*, indicating that it serves as an antioxidant defense against oxidative stress [81]. MDHAR activity decreases under Cd stress, although *Brassica*’s antioxidant defense mechanism is bolstered when hydrogen peroxide is provided beforehand [71]. Additionally, mustard plants under Pb stress exhibit increased MDHAR activity when salicylic acid (SA) is added, strengthening the plants’ antioxidant defenses and fostering better growth [82]. Additionally, exogenous EDTA delivery to mustard seedlings under Cd stress enhances the components of the AsA-GSH cycle, especially MDHAR, which reduces oxidative damage and promotes development by limiting Cd uptake and increasing the concentration of nonprotein thiols [83]. It has also been shown that MDHAR is involved in maintaining the pool of reduced ascorbate at its optimum level, in recycling the oxidized ascorbate, and in regulating the redox balance to scavenge the ROS when the cells are exposed to various stress agents [84]. Research has demonstrated that MDHAR genes are essential elements of the antioxidant defence system, supporting the improvement of antioxidant scavenging systems and the plants’ overall ability to withstand stress in *Brassica* varieties [85]. *Brassica rapa*’s increased resistance to freezing stress has been associated with the expression of MDHAR genes, and co-expression of MDHAR and DHAR genes has been shown to boost stress tolerance mechanisms [86].

### 3.6. Dehydroascorbate Reductase (DHAR)

Dehydroascorbate reductase, or DHAR, is an important enzyme that helps cells regenerate ascorbate (AsA), which lowers oxidative stress in cells [87]. Excessive DHAR synthesis raises the pace at which AsA regenerates and initiates the Ascorbate-Glutathione Cycle, which scavenges ROS in the presence of intense light [88]. In *Brassica* plants, DHAR is an essential component of defense systems against environmental stressors, especially heavy metal toxicity, such as Cd [89].

Through the regeneration of ascorbate, a vital antioxidant, DHAR effectively preserves plants’ capacity to neutralize ROS [90]. By lowering oxidative damage, it improves *B. juncea*’s antioxidative defenses under zinc stress [91]. When subjected to Cd stress, *B. juncea*, a plant that is relatively resistant to Cd toxicity, exhibits a sharp increase in DHAR activity [92]. Together with GR and MDHAR, this strengthens antioxidant defense systems [93]. DHAR’s role in linking the ascorbate and glutathione pools with H_2_O_2_ metabolism is crucial for plant defense, growth, and development [94]. This underlines DHAR role in decreasing the oxidative damage and enhancing resistance to the stress factors in *Brassica* species.

### 3.7. Glutathione Peroxidase (GPX)

GPX plays a key role in *Brassica* defense mechanisms against various stressors. According to research done on rapeseed, the GPX genes are crucial for regulating stress, ROS, and antioxidant processes [95]. Additionally, the role of glutathione in controlling the miRNA synthesis during pathogen attack is demonstrated using the model plant *Arabidopsis thaliana*. *Alternaria brassicicola* targets defense-related genes, kinases, and transcription factors, which in turn improve resistance to infection [96]. Higher levels of GSH were found to confer resistance against necrotrophic infections. Additionally, fungal infections in winter oilseed had a substantial impact on GPX activity [97]. *Alternaria brassicicola* was the source of the largest increase in GPX activity, indicating the importance of GPX in defense responses. Furthermore, *Brassica* species can enhance GPX in response to selenium (Se) exposure, including stress tolerance and Se-dependent GPX activity, which is advantageous for phytoremediation applications [98]. Because it promotes antioxidant defense, stress response, and detoxification pathways, GPX is crucial for *Brassica* defense against heavy metal stress and pathogen incursions [99].

## 4. Glucosinolates in *Brassica* spp.

**Figure 2:**
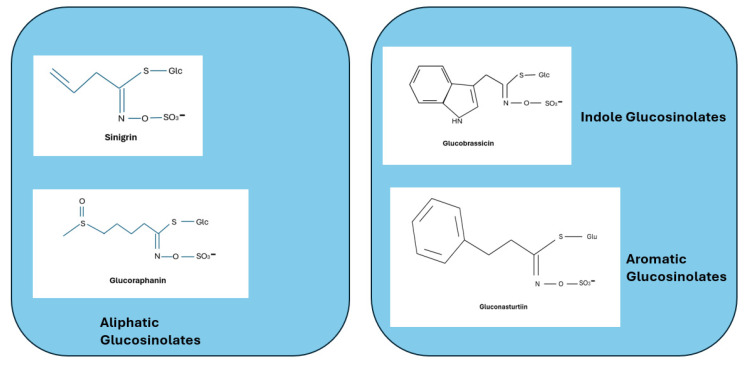
Types of glucosinolates found in *Brassicaceae*.

Glucosinolates (GLs), which are prevalent in *Brassica* vegetables, have several health benefits. They are substances that contain nitrogen and sulfur. It has also been demonstrated that they have certain health benefits, such as the ability to prevent the development of cancer by preventing the production of metabolites [100]. Key enzymes like sulfotransferases catalyze a sequence of reactions that change amino acids, which can start the manufacture of GSLs [101]. The structure of the glucosinolate consists of a β-thioglucose moiety, a sulfonated oxime moiety, and a variable aglycone side chain derived from a α-amino acid (Figure 2).

**Figure 3:**
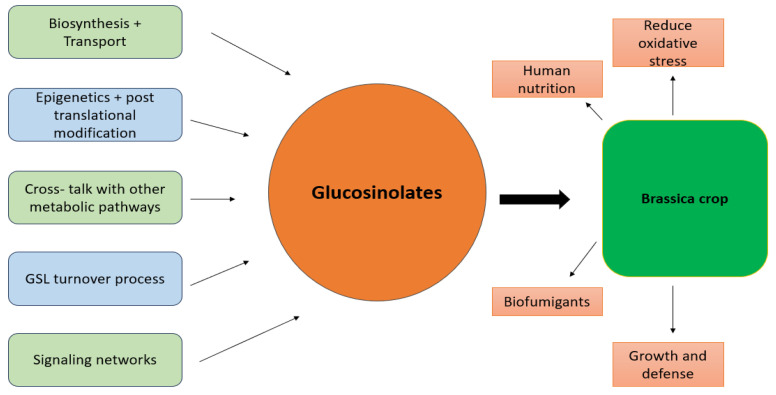
Glucosinolates activation and effect on *Brassica*.

In *Brassica rapa*, 102 putative GLs biosynthetic genes were identified, showing high co-linearity with *Arabidopsis*, indicating conserved pathways [102]. The last stages of GLs biosynthesis include the addition of sulphate groups, which are important in the formation of the active compounds [103]. In silico research, which forecasts interactions with antioxidant enzymes, indicates that they possess antioxidant properties. These compounds are also found in *Brassica* oilseeds, where they improve flavor. However, because excessive levels of these molecules might be dangerous, they must be eliminated [104]. Although agronomic and environmental factors can cause significant variations in concentrations, with some compounds showing differences of up to 556 times, genetic factors are the primary determinants of the glucosinolate profiles of *Brassica* crops [105]. Furthermore, the amount of glucosinolate varies depending on the type of *Brassica* and how it is prepared for ingestion, which always reduces the plant’s potential to promote health. Some recent studies have examined the potential health advantages of glucosinolates, which are thought to be beneficial after demonstrating the chemicals’ capacity to shield cells from oxidative stress [73] (Figure 3). A higher glucosinolate content in *Brassica* spp. has been associated with improved resistance to oxidative stress brought on by external stimuli. For instance, research indicates that plants with higher glucosinolate levels are more resilient to oxidative damage during periods of drought or pathogen-induced stress [16]. This is probably because their breakdown products have a protective function.

*Brassica* plants can generate glucosinolates as a secondary metabolic reaction to oxidative stress. By lowering ROS accumulation and triggering oxidative stress response pathways, including the Nuclear Factor erythroid 2-related factor 2 (Nrf2) and the Antioxidant Response Element (ARE) pathway, these chemicals activate the plant’s stress defense mechanisms through their breakdown products. By increasing antioxidant enzyme activity, decreasing reactive oxygen species, and modifying oxidative stress response pathways in *Brassica* plants and animals that eat them, GL breakdown products like sulforaphane aid in the reduction of oxidative stress [106]. Metabolites obtained by the hydrolysis of consumed GLs possess antibacterial, anti-inflammatory, and anticarcinogenic effects. GL breakdown products have been related to upregulating the Nrf2 protein, which has neuroprotective effects and holds promise in treating diabetes, cancer, and cardiovascular illnesses, as sulforaphane (SFN) has nutraceutical properties as well. Furthermore, by causing apoptosis, generating ROS, and blocking signaling cascades like NF-κB and ERK (Extracellular Signal-Regulated Kinase) in colorectal carcinoma cells, *B. rapa* with high GL has demonstrated cancer-preventive properties [107]. Considering all of the facts, it is evident that eating foods high in GL has several health benefits, placing them in a category for active and preventive nutrition [108]. In particular, there is a need to better understand the genetic and environmental factors affecting glucosinolates in *Brassica* plants to develop higher value products with enhanced pharmacological activity [39].

## 5. Pattern of Activity of Glucosinolates to Regulate Enzymatic Activity

Glucosinolates, as a class of secondary metabolites found in *Brassica*, are crucial for controlling the activity of enzymes. Research indicates that glucosinolates are hydrolyzed by the natural enzyme myrosinase to produce a range of compounds, including isothiocyanates that are beneficial to health [109]. Broccoli and cabbage contain Glucosinolates, which give these vegetables their unique tastes and health benefits [110]. They also promote the ability of plants to defend themselves. In addition, myrosinase-catalyzed enzymatic reactions are essential for the formation of glucosinolate derivatives as products with anti-inflammatory and anti-cancer properties [111]. Understanding glucosinolate metabolism is crucial to maximizing the health benefits of consuming *Brassica* vegetables because a number of variables, such as growing conditions, cabbage morphotype, and accession, may impact the bioavailability of these advantageous compounds [112].

**Figure 4:**
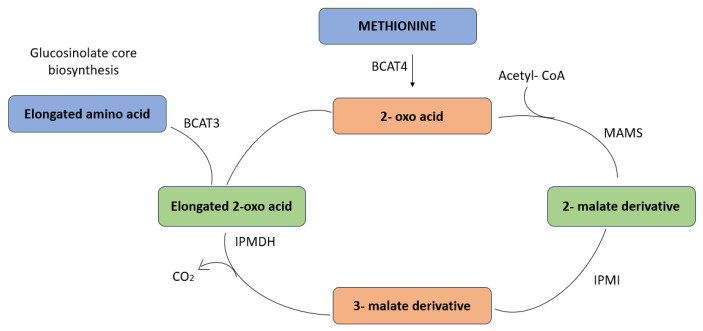
Glucosinolate chain elongation.

When the branched-chain aminotransferase 4 (BCAT4) catalyzes the conversion of methionine to its corresponding 2-oxo acid, 4-(methylsulfanyl)–2–oxobutanoate, the chain elongation cycle begins. Isopropylmalate dehydrogenase (IPMDH), isopropylmalate isomerase (IPMI), and methylthioalkylmalate synthase (MAMS) mediate a series of processes that lengthen the aliphatic chain by one methylene group. BCAT3 has the ability to either transaminate the extended 2-oxo to the proper amino acid or reenter the Chain Elongation Cycle (Figure 4).

**Figure 5:**
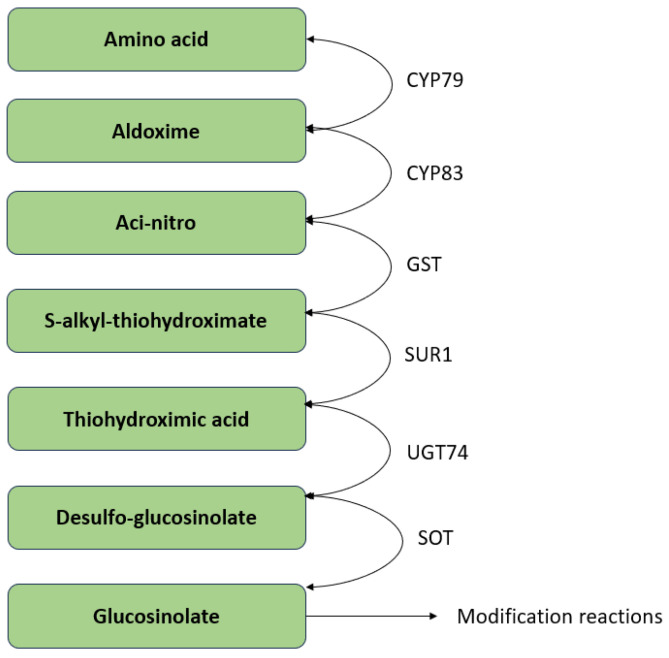
Biosynthesis of core glucosinolates.

Members of the CYP79 cytochrome P450 family may convert a range of amino acids (i.e., variable R-group), including extended aliphatic methionine-derived compounds (Figure 5), to aldoximes, which can then be used to start building the core glucosinolate scaffold. The unstable aci-nitro compounds produced by members of the CYP83 family are thought to serve as the substrate for glutathione-S-transferases, which are responsible for introducing the shared sulfur atom. The S-alkyl-thiohydroximate is changed into thiohydroximic acid by the SUR1 cysteine-sulfur lyase. Finally, UDP-glucosyltransferase (UGT) family 74 enzymes are used to attach a glucosyl residue to the modified acid. The final step is catalysis by PAPS-dependent sulphotransferases (SOT) in sulfation. The molecular variety of glucosinolates is the result of subsequent alteration reactions of the basic structure.

**Figure 6:**
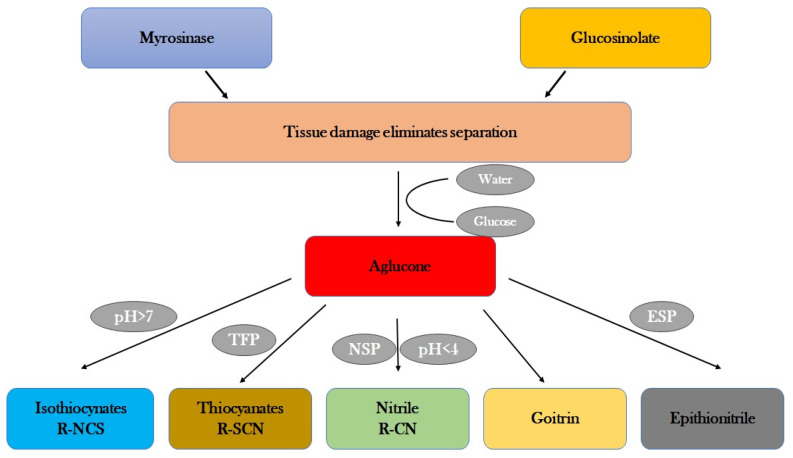
Glucosinolates and regulation of enzymatic activity.

Because they alter antioxidant enzymes through the Keap1-Nrf2-ARE pathway, glucosesinolates are significant. Heme Oxygenase-1 (HO-1), Glutamate-Cysteine Ligase Catalytic subunit (GCLC), Glutathione S transferases (GSTs), and NAD(P)H quinone reductase (NQO1) are further examples of these enzymes [113]. The ability of these bioactive compounds, especially the hydrolysis products of aliphatic isothiocyanate glucosinolate, to repel a range of biotic threats and exhibit chemopreventive properties in mammalian systems—including the ability to prevent cancer by controlling antioxidant enzymes—is well known [114]. In addition, MYB and basic helix-loop-helix factors interact in rather complex transcriptional networks that control the biosynthesis of glucosinolates [115]. These factors work in concert with phytohormones, such as Jasmonate, to allow coordinated and rapid control of glucosinolate genes [116]. The intricate relationships among transcriptional regulation, enzymatic activity, and glucosinolates in plant defense (Figure 6) and the potential benefits to human health are illustrated by these techniques. GSLs regulate the activity of several enzymes involved in various biological processes and are found in *Brassica* species. Recent studies reveal candidate genes associated with GSL biosynthesis, including BnaMAM1, BnaGGP1, BnaSUR1, BnaMYB51, BnaMYB44, BnaERF025, BnaE2FC, BnaNAC102, and BnaDREB1D, that act as regulators of enzyme activity [117]. Additionally, GSLs have shown that they can alter enzyme activity in *Brassica* species to provide a variety of physiological advantages, such as resistance to pests and diseases, as well as allelopathic and anticarcinogenic qualities [118]. It draws attention to the intricate relationship between GSLs and the regulation of enzymes in *Brassica* crops, showing how genetic and environmental variables determine the variance in GSL content in *Brassica*, which ultimately affects enzyme activity and health-promoting properties [119]. Temperature, light, infections, and other environmental stimuli cause different chemicals to be produced, which in turn affects GSL biosynthesis and gene expression [120]. Thus, through eATP (Extracellular ATP), the indolic glucosinolate pathway is induced upon an attack from pathogens or herbivores to enhance plant defence against a wide range of pathogens or herbivores [121]. Additionally, glucosinolates degrade myrosinases to produce toxic substances that shield the plant from diseases and create a defense mechanism between glucosinolates and myrosinase [122]. Additionally, Nitrile-Specifier Proteins (NSPs) and Epithiospecifier Proteins (ESPs) in certain plants, such as cabbage, aid in rerouting glucosinolate hydrolysis (Figure 6), providing plants with an extremely powerful defense mechanism against pests [123].

## 6. Regulation of Enzymatic Activity in Response to Oxidative Stress

Heme oxygenase 1 (HO-1), glutathione S transferases (GSTs), and NAD(P)H quinone reductase (NQO1) are examples of Nrf2 signaling pathway enzymes that play crucial roles in shielding cells from oxidative stress. Furthermore, it has been discovered that isothiocyanates bind to regulators of oxidative stress responses in fungal cells, potentially having physiological importance. This finding may have implications for adaptation to a variety of stressors [124,125]. Nevertheless, such compounds enhance antioxidant defenses normally, but could be damaging through oxidation in certain circumstances, such as through phase-I enzymes, thus implying heightened carcinogenic potential in some circumstances. Therefore, the degradative products of glucosinolates are capable of both antioxidant and pro-oxidative effects and modulate oxidant-antioxidant balance at the cellular level, always in the biological systems. Enzymes are subjected to regulation mechanisms to facilitate their adaptation to oxidative stress and maintain integrity within the cells. The enzymes influenced by oxidative stress are nucleoside diphosphatases (NDPases), aldehyde dehydrogenases (ALDHs), and glyceraldehyde-3-phosphate dehydrogenase (GAPDH) [126]. Sulfhydryl group reactions, for instance, control GAPDH and alter the protein’s enzymatic function and non-glycolytic activity in both reversible and irreversible ways [127]. The selective inhibition of ALDHs in response to oxidative stress reroutes the carbon supply to fulfill the demands of the cell [128]. The glycosylation of proteins may also be affected by ROS’s effect on NDPases. NDPases are used to mature glycoproteins [40]. These outcomes elucidate the multiple regulatory mechanisms that enzymes utilise to down-regulate oxidative stress and sustain biological functions. Therefore, enzymes such as CAT, SOD, POX, GPX, and others constitute a complex antioxidant defence to eliminate ROS efficiently and to decrease oxidative stress.

## 7. Conclusions

*Brassica* species, including *B. oleracea, B. napus*, and *B. juncea*, are essential to agriculture and human nutrition. They are rich in minerals, antioxidants, and bioactive substances that are essential for maintaining good health and a balanced diet. However, oxidative damages resulting from abiotic factors, such as environmental stress and heavy metals, poses a risk to these crops. Oxidative stress causes cellular damage and reduces agricultural yield in *Brassica* plants by upsetting the balance between antioxidant defense processes and ROS production. *Brassica* species have a well-coordinated enzymatic defence system that involves GR, SOD, CAT, APX, MDHAR, DHAR, and GPX. These enzymes neutralize ROS. Redox equilibrium is essential for plant growth and development under stress. Glucosinolates, which are compounds found mainly in Brassicaceae, have a multifaceted function in decreasing oxidative stress in plants through their ability to act as antioxidants. When plant tissue is damaged, glucosinolates are decomposed into bioactive chemicals, like isothiocyanates, which can directly neutralize free radicals and stimulate antioxidant protection in plant cells. It improves the plant’s defense against oxidative damaging agents through the use of antioxidant pathways, including Keap1-Nrf2-ARE. It is necessary to have a basic grasp on how GLs and the associated enzyme defense system vary in the genetic background and in reaction to environmental conditions in order to create new *Brassica* genotypes with improved stress tolerance and nutritional value. The primary goal of future research should be to maximize these natural defenses to guarantee sustainable agriculture.

## Data Availability

All data are included in the manuscript.
